# Supplementary material for: The Time-Resolved Salt Stress Response of Dunaliella tertiolecta—A Comprehensive System Biology Perspective
Source: Int J Mol Sci. 2023 Oct 19;24(20):15374. doi: 10.3390/ijms242015374 (PMC10607294; doi:10.3390/ijms242015374)
Supplement: Supplementary file 1 [file ijms-24-15374-s001.zip › Supplements Figure.pdf]

Supplements

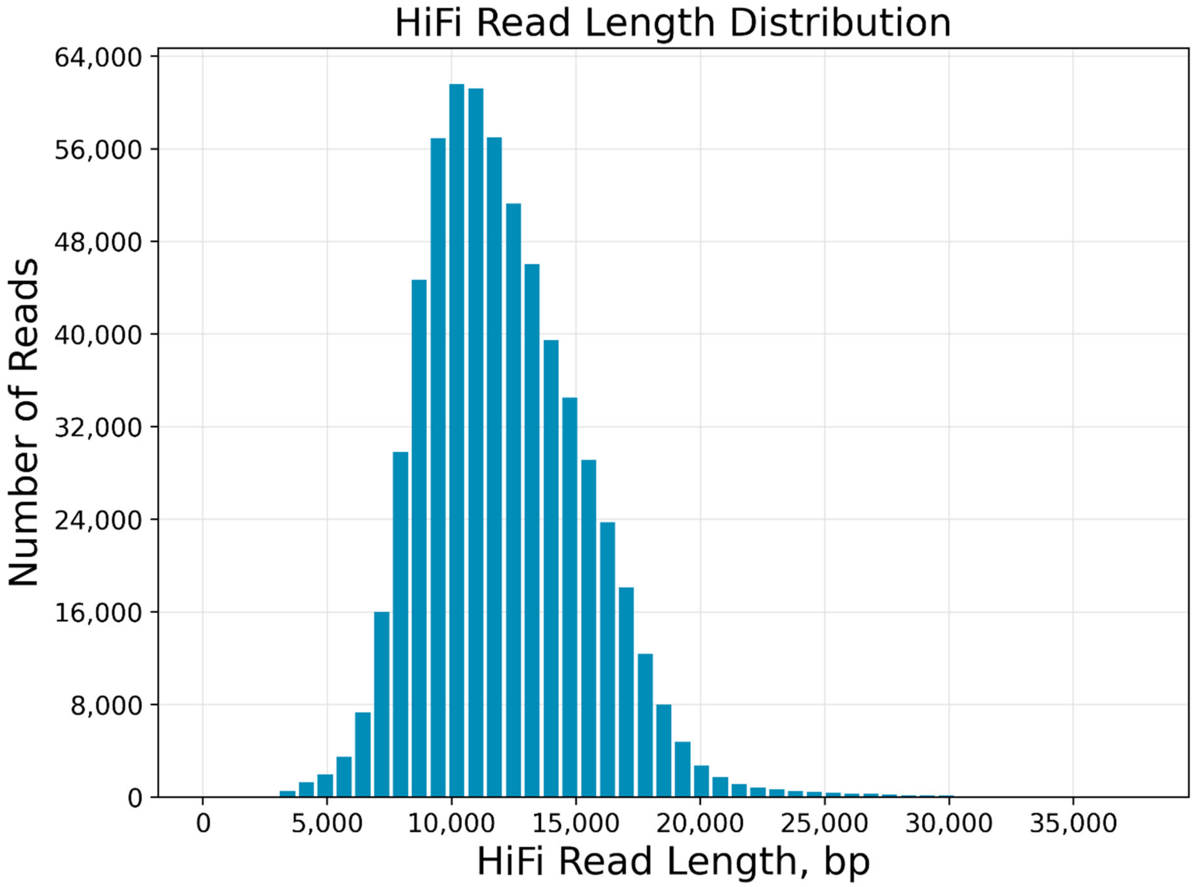

Figure S1. HiFi Read Length Distribution filtered (>Q20) genome sequence data from *D. tertiolecta*

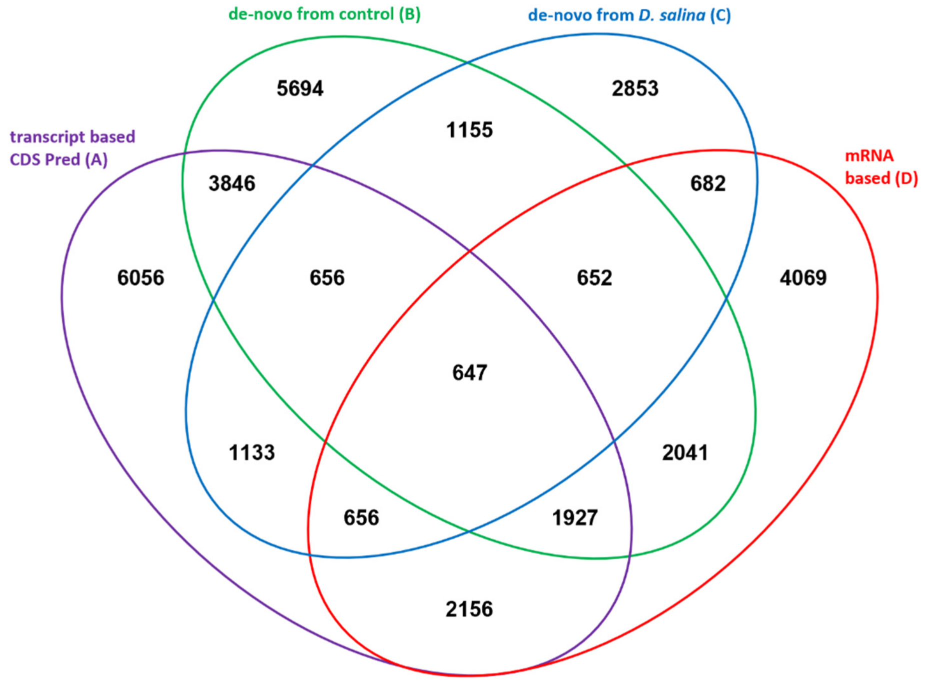

Figure S2. Venn diagram of identical predicted genes from *D. tertiolecta* after removing redundant protein coding sequences that have been identified in all proteomics data sets using PEAKS with SPIDER. A: set of all *de-novo* predicted genes from 2 M NaCl for 30 min and 4 h treated cells and the control without NaCl using Augustus-BRAKER. B: *de-novo* predicted genes using the predicted Augustus-BRAKER training set from the 0 M NaCl sample. C: *de-novo* predicted genes using the available Augustus training set for *Dunaliella salina*. D: predicted open reading frames from assembled mRNA data from all transcripts (2 M NaCl for 30 min and 4 h treated cells and the control without NaCl).

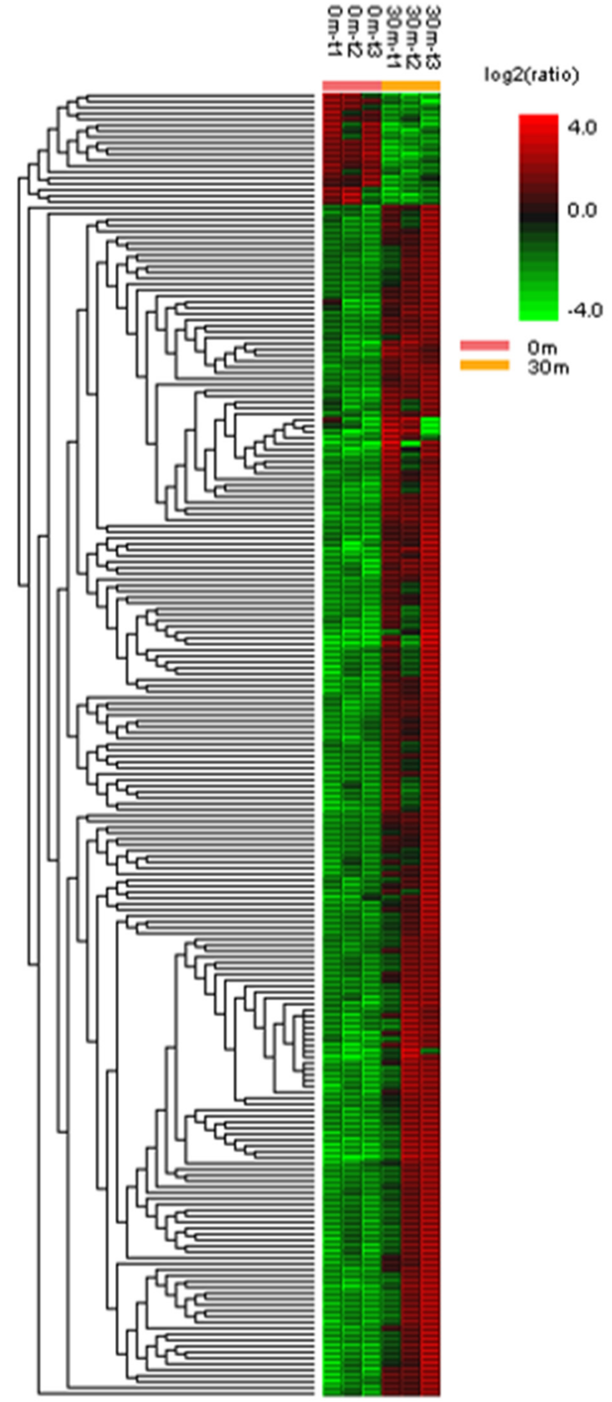

Figure S3. Heatmap of up- or down-regulated proteins after 30 min of salt stress.

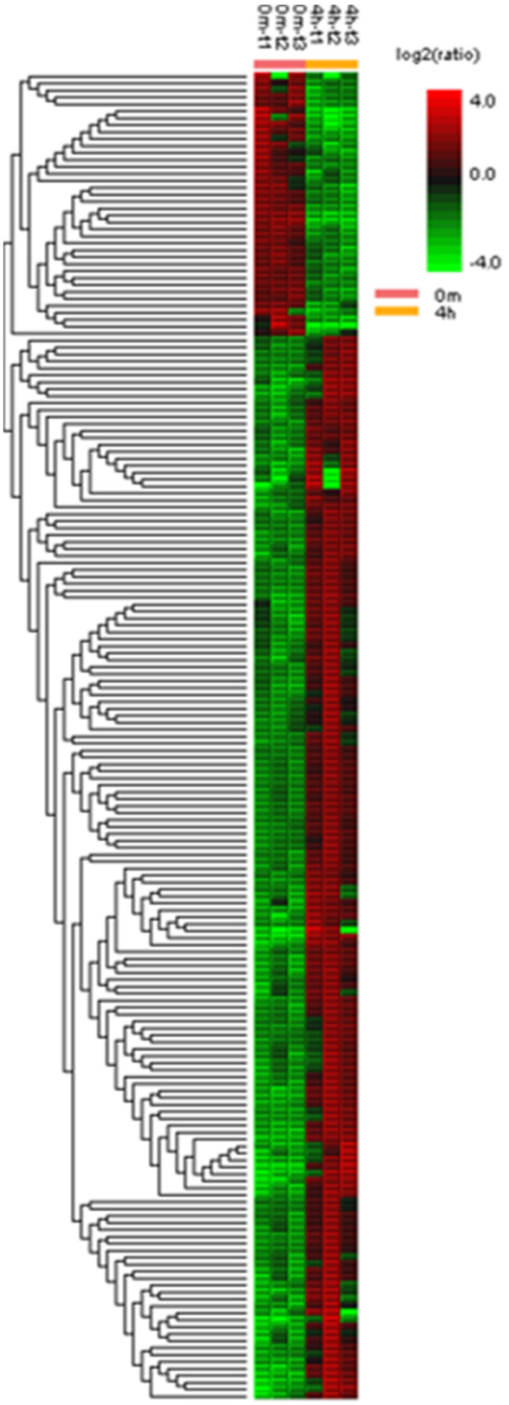

Figure S4. Heatmap of up- or down-regulated proteins after 4 h of salt stress.

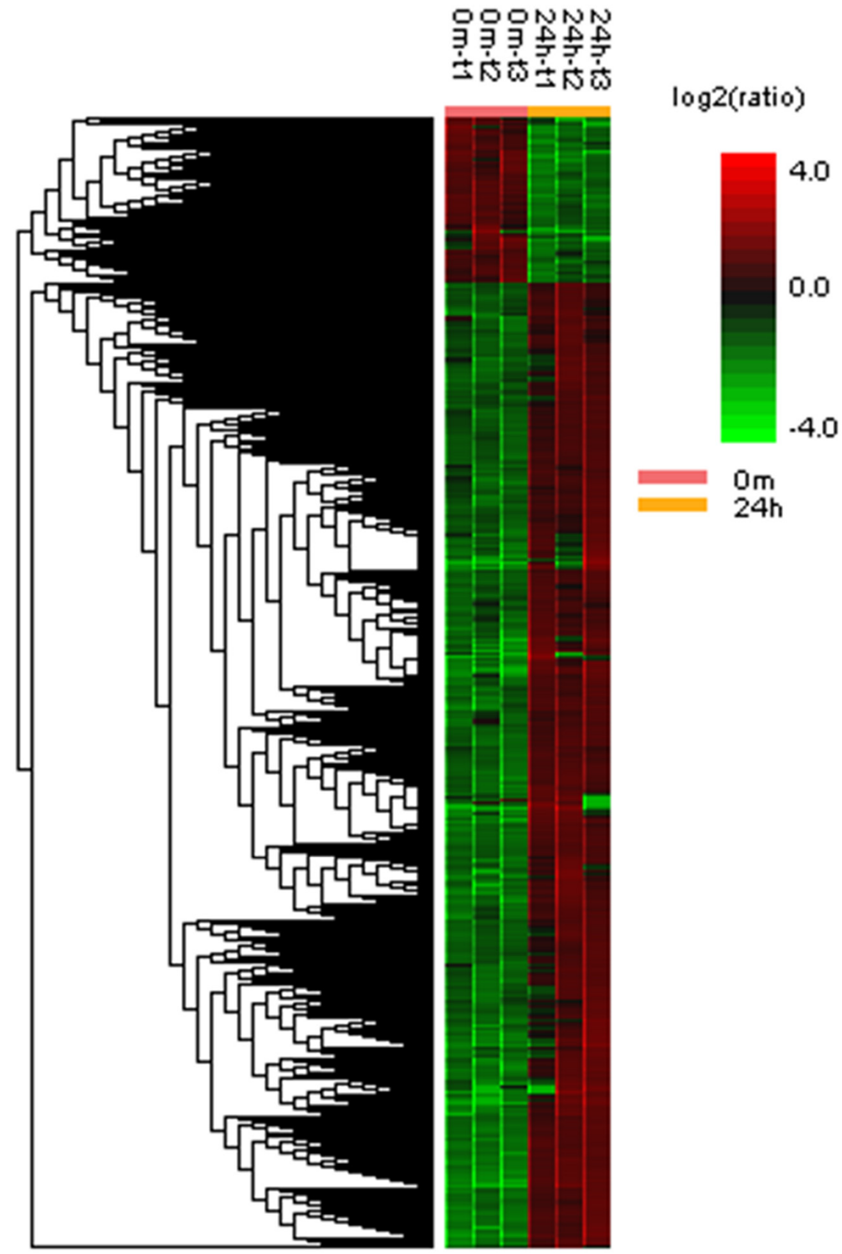

Figure S5. Heatmap of up- or down-regulated proteins after 24 h of salt stress.
